# Global research trends in pediatric bone and joint infections: A 50-year bibliometric analysis (1976–2025)

**DOI:** 10.1051/sicotj/2026024

**Published:** 2026-05-27

**Authors:** Raju Vaishya, Ashok N Johari, Brij Mohan Gupta, Ghouse Modin Nabeesab Mamdapur, K.S. Ali, Abhishek Vaish

**Affiliations:** 1 Indraprastha Apollo Hospitals Sarita Vihar New Delhi 110076 India; 2 Childrens’ Orthopaedic Centre, Bobby Apartments Lady Jamshedi Roda, Mahim Mumbai 400016 India; 3 Retired Scientist from CSIR-NISTADS, Pusa New Delhi 110012 India; 4 Yenepoya (Deemed to be University), Department of Library and Information Science, Deralakatte Mangalore 575018 Karnataka. India; 5 St. Joseph’s University Bengaluru 560027 Karnataka India; 6 Indraprastha Apollo Hospitals, Sarita Vihar New Delhi 110076 India

**Keywords:** Pediatric bone infections, Septic arthritis, Bibliometric analysis, Highly cited publications, International collaboration

## Abstract

*Background*: Pediatric bone and joint infections (PBJI) remain a major cause of morbidity in children, with evolving trends in epidemiology, diagnostics, and management. A comprehensive understanding of the global research landscape is essential to identify influential contributions, collaboration patterns, and thematic priorities. This study aimed to perform a bibliometric analysis of PBJI research to map productivity, impact, and knowledge structure. *Methods*: A systematic literature search was conducted in a major bibliographic database to retrieve PBJI-related publications from 1976 to 2025. Bibliometric indicators including total publications (TP), total citations (TC), citations per paper (CPP), relative citation index (RCI), highly cited papers (HCPs), and international collaborative papers (ICPs) were analyzed. Network analyses of keywords, authors, institutions, countries, and journals were performed using VOSviewer to assess collaboration patterns and research themes. *Results*: A total of 1,556 publications were identified. Journal articles dominated output (82.4%), while reviews showed higher impact (CPP 31.67; RCI 1.90). English-language papers accounted for 89.1% of publications and 97.5% of citations, including all 28 HCPs. Research output and impact were concentrated in a few high-income countries, led by the USA and the UK. Citation distribution was highly skewed, with 18.4% uncited papers and only 6.3% of publications receiving more than 50 citations. Keyword analysis revealed four major thematic clusters centered on osteomyelitis, septic arthritis, pathogens (especially *Staphylococcus aureus*), diagnostics, and treatment. Collaboration networks were selective and fragmented, with a small core of influential authors and institutions. *Conclusion*: PBJI research is a mature but uneven field, driven by a limited number of high-impact contributors and focused clinical themes. Strengthening international collaboration and improving visibility of research from underrepresented regions are critical for balanced global advancement.

## Introduction

Pediatric bone and joint infections (PBJI) represent a global health challenge, comprising acute hematogenous osteomyelitis, septic arthritis, and associated complications in children under 18 years. These infections are associated with high morbidity, including growth plate disturbances, limb deformities, and chronic sequelae, affecting thousands annually worldwide.

In low- and middle-income countries (LMIC), delayed diagnosis due to limited resources exacerbates outcomes, while in high-resource settings, emerging pathogens and antimicrobial resistance pose ongoing threats [1]. Globally, the incidence of PBJIs is estimated at 8–10 per 100,000 children, with peaks in those aged 1–5 years, driven by hematogenous spread from transient bacteremia [2]. Chronic cases often require multidisciplinary interventions, including surgical debridement, targeted antibiotics, and rehabilitation, emphasizing the urgency for evidence-based prevention and management strategies tailored to pediatric physiology.

Despite advances in molecular diagnostics, Polymerase Chain Reaction (PCR) for pathogen detection, and early imaging, gaps remain in identifying subtle presentations, such as those caused by fastidious organisms *like Kingella kingae*, and in optimizing antibiotic stewardship to minimize resistance [3].

Highly cited publications (HCPs) are instrumental in informing pediatric guidelines and research directions, spotlighting influential studies that advance orthopedics and pediatric infectious diseases [4]. Bibliometric analyses provide a robust framework to quantify scientific productivity, delineate thematic clusters, and predict trends. Prior bibliometric efforts have examined citation classics in pediatric orthopedics and other subjects [5–7] or specific PBJIs like neonatal osteomyelitis, highlighting themes in microbiology and imaging, with journals like *Journal of Bone and Joint Surgery* (JBJS) predominating [2, 8]. Yet, a holistic global bibliometric assessment of HCPs on PBJI is lacking, especially one encompassing diverse etiology from acute to subacute forms across age groups.

The PBJI’s publication uptick post-2010 was propelled by advances in *K. kingae* diagnostics and oral antibiotic trials, with landmark papers on CRP-based prediction rules and PCR identification as citation leaders [9, 10]. This analysis benchmarks the PBJI knowledge ecosystem and guides future priorities for prioritizing accessible innovations for global equity in this area [11, 12]. By mapping the scholarly terrain of PBJI research, this bibliometric examines the published research in PBJI, including the high-impact publications, which may have a profound impact on these infections and child health [13, 14].

## Materials and methods

### Data source and search strategy

This bibliometric study analyzed global research on PBJI using the Scopus database, accessed on 3 January 2026. Scopus was chosen for its broad journal coverage, standardized metadata, and robust citation indexing. A comprehensive keyword-based search strategy targeted osteomyelitis and septic arthritis in pediatric populations, combining disease-specific terms, child and adolescent descriptors, and Scopus age-related keywords, while excluding adult-focused records. Boolean operators and truncation were used to maximize sensitivity. Publications from 1976 to 2025 were included, with no language restrictions. The search string for the study is presented in Supplementary Box 1.

### Sampling strategy and data extraction

The final dataset comprised 1,366 records. A purposive sampling strategy retained all eligible publications to ensure comprehensive coverage over the 50 years. Complete bibliographic metadata was exported in CSV format in a single session to avoid database inconsistencies.

### Analysis of bibliometric indicators and variables

Bibliometric indicators assessed research productivity, impact, and collaboration patterns, including total publications (TP), total citations (TC), citations per paper (CPP), relative citation index (RCI), temporal trends, geographic distribution by country and continent, journal productivity, authorship patterns, and international collaborative papers (ICP). Highly-cited papers (HCPs) were those that which received 100 or more total citations.

### Network mapping and visualization

Network analyses were conducted using VOSviewer (version 1.6.20) to map co-authorship, keyword co-occurrence, and co-citation networks, revealing collaboration structures, thematic clusters, and the intellectual foundations of the literature. Association strength normalization and default visualization settings were applied for transparency and comparability.

### Data analysis

Descriptive and supplementary statistical analyses were performed using Microsoft Excel to generate publication trends, citation metrics, and geographic summaries. Data validation procedures included duplicate checks and standardized documentation of all methods, tools, and parameters to ensure rigor, reproducibility, and adherence to best practices in bibliometric research.

## Results

### Temporal trends in output and impact

**Figure 1:**
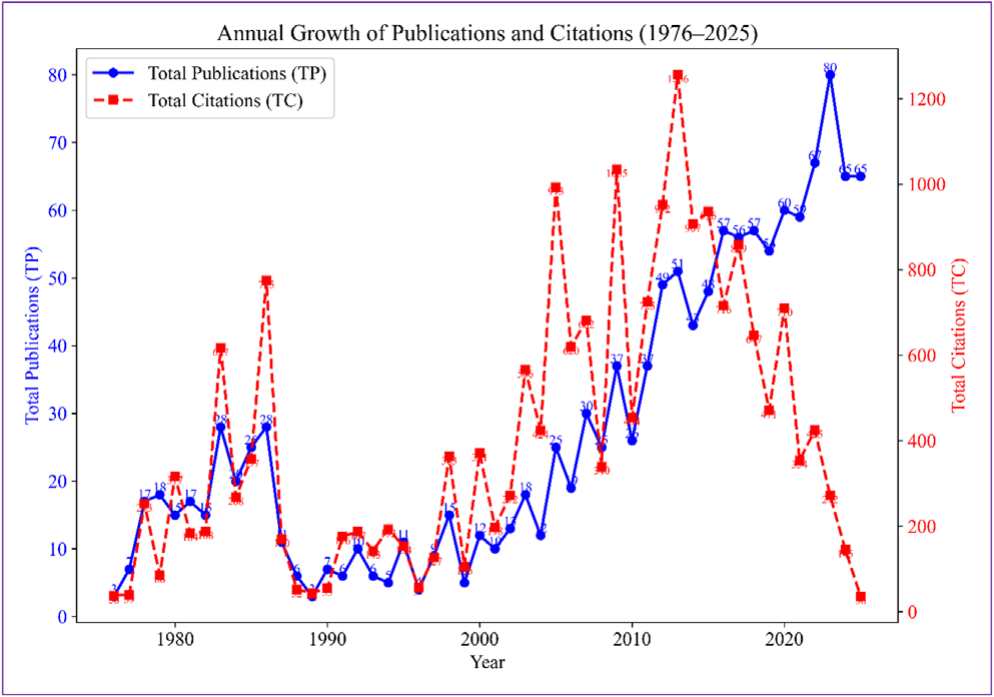
Five-year slab publications growth and citations trend (1976–2025).

The year-wise analysis shows a sustained expansion of PBJI research from 1976 to 2025 (Supplementary Table 1 and Figure 1). Overall, 1,366 publications received 20,315 citations (CPP = 14.87; RCI = 1.00). Early years were characterized by low publication output but relatively high citation impact, with occasional peaks in CPP and RCIs above unity. From the mid-1980s to early 2000s, the field matured, with steady growth in output, the emergence of highly cited papers (HCPs), and several high-impact years, notably 2005. After 2010, publication volume and team size increased sharply. However, this quantitative growth was accompanied by declining per-paper impact, with CPP and RCI decreasing substantially in recent years. International collaboration remained limited overall, despite sporadic peaks in certain years. In contrast, funded research increased markedly in the last decade, indicating stronger institutional and policy support, even though recent publications have accrued fewer citations to date.

Figure 1 shows five-year aggregated trends indicating a steady rise in publication output in PBJI research from 1976 to 2025, with especially rapid growth after 2000. In contrast, total citations peak in the mid-2000s and decline in more recent periods, reflecting the shorter time available for newer publications to accumulate citations.

### Document types

Research articles dominate PBJI research, contributing 1,125 publications (82.4%) and 15,156 citations, but with only moderate impact (CPP = 16.44) and a below-average RCI (0.91). In contrast, reviews, though fewer (156 papers; 11.4%), show substantially stronger influence, accruing 4,402 citations with a CPP of 31.67 and RCI of 1.90, and accounting for 9 of 28 HCPs. Short surveys, despite just seven publications, demonstrate the highest impact (CPP = 45.29; RCI = 3.05). Other formats (letters, notes, editorials, conference papers) contribute minimally, with low CPPs (2.67–19.00) and RCIs below one. International collaboration is modest overall, higher in reviews (12.18% ICP) than in research articles (9.24%) (Supplementary Table 2).

### Language-wise distribution

English dominates PBJI research with 1,217 publications (89.1%) and 19,803 citations (97.5%), achieving a CPP of 19.19 and RCI of 1.09; all 28 HCPs are in English. Other languages show markedly lower impact, including French (50 papers; CPP 5.65; RCI 0.24), German (32 papers; CPP 9.09; RCI 0.42), Spanish (CPP 7.11; RCI 0.24), and Russian (CPP 2.20; RCI 0.04). Minor languages contribute few papers with occasional higher CPPs (e.g., Portuguese CPP 12.00), but overall citation visibility remains limited outside English (Supplementary Table 3).

### Citation distribution and concentration of research impact

Citation impact is highly skewed: 251 papers (18.4%) remain uncited, while 616 papers (45.1%) receive only 1–10 citations (CPP = 4.18). Although fewer in number, papers cited 11–25 times (261) and 26–50 times (152) contribute substantially, with CPP rising to 16.89 and 35.59, respectively. High-impact output is concentrated in just 86 papers (6.3%) with over 50 citations, showing CPPs above 70 and reaching 253.5 for papers cited more than 200 times (Supplementary Table 4).

### Country-wise research output, collaboration, and citation impact

Eighty countries contributed to 1,556 publications, with output highly skewed: most countries produced fewer than 10 papers, while only the USA (389) and UK (106) dominated. The USA leads with 389 publications, 7,963 citations (CPP 20.47; RCI 1.38), 14 HCPs, and 12.85% international collaboration. The UK, France, and Germany show moderate output (73–106 papers) with good impact (CPP ~17–18; RCI >1.0). India (93 papers) and China (76 papers) contribute substantial output but low impact (CPP <8.0; RCI <0.6); China has strong funding (50 papers) but minimal collaboration (2.63% ICP). Smaller contributors such as Canada and Belgium achieve high impact (CPP >24; RCI >1.6) with strong collaboration, while Switzerland shows the highest collaboration rate (50% ICP) with moderate impact. Table 1 presents the bibliometric profile of the top 20 countries.

**Table 1:** Most productive and collaborative countries.

Country	TP	TC	CPP	TA	HCP	FP	ICP	%ICP	RCI	Links
United States	389	7963	20.47	1608	14	33	50	12.85	1.38	74
United Kingdom	106	1836	17.32	466	3	11	28	26.42	1.16	45
France	93	1627	17.49	533	5	9	21	22.58	1.18	33
India	93	641	6.89	403	0	11	12	12.90	0.46	24
China	76	594	7.82	445	0	50	2	2.63	0.53	2
Germany	73	1332	18.25	443	2	12	16	21.92	1.23	36
Italy	58	755	13.02	453	0	14	16	27.59	0.88	29
Canada	47	1153	24.53	269	3	9	19	40.43	1.65	36
Spain	43	469	10.91	382	0	4	12	27.91	0.73	34
Japan	42	522	12.43	299	1	10	4	9.52	0.84	8
Turkey	35	524	14.97	185	1	2	4	11.43	1.01	4
Switzerland	32	485	15.16	205	0	8	16	50.00	1.02	25
Israel	32	480	15.00	186	0	1	8	25.00	1.01	13
Australia	31	549	17.71	135	0	5	9	29.03	1.19	12
South Korea	29	492	16.97	150	0	4	4	13.79	1.14	5
Belgium	22	533	24.23	105	2	4	7	31.82	1.63	19
New Zealand	20	205	10.25	99	0	8	6	30.00	0.69	14
Netherlands	20	199	9.95	143	0	5	7	35.00	0.67	17
Egypt	18	217	12.06	96	0	1	7	38.89	0.81	12
Poland	17	186	10.94	79	0	2	3	17.65	0.74	6
Top 20 Countries	1276	20762	16.27	6684	31	203	251	19.67	1.00	448
Other 60 Countries	280	3278	11.71	1450	4	44	76	27.14	1.00	134
Total 80 Countries	1556	24040	15.45	8134	35	247	327	21.02	1.00	582

TP = Total Publications; TC = Total Citations; CPP = Citations per Paper; TA = Total Authors; HCP = Highly cited papers; FP = Funded Papers; ICP= International Collaborative Papers; RCI = Relative Citation Index.

### Continental contributions

**Figure 2:**
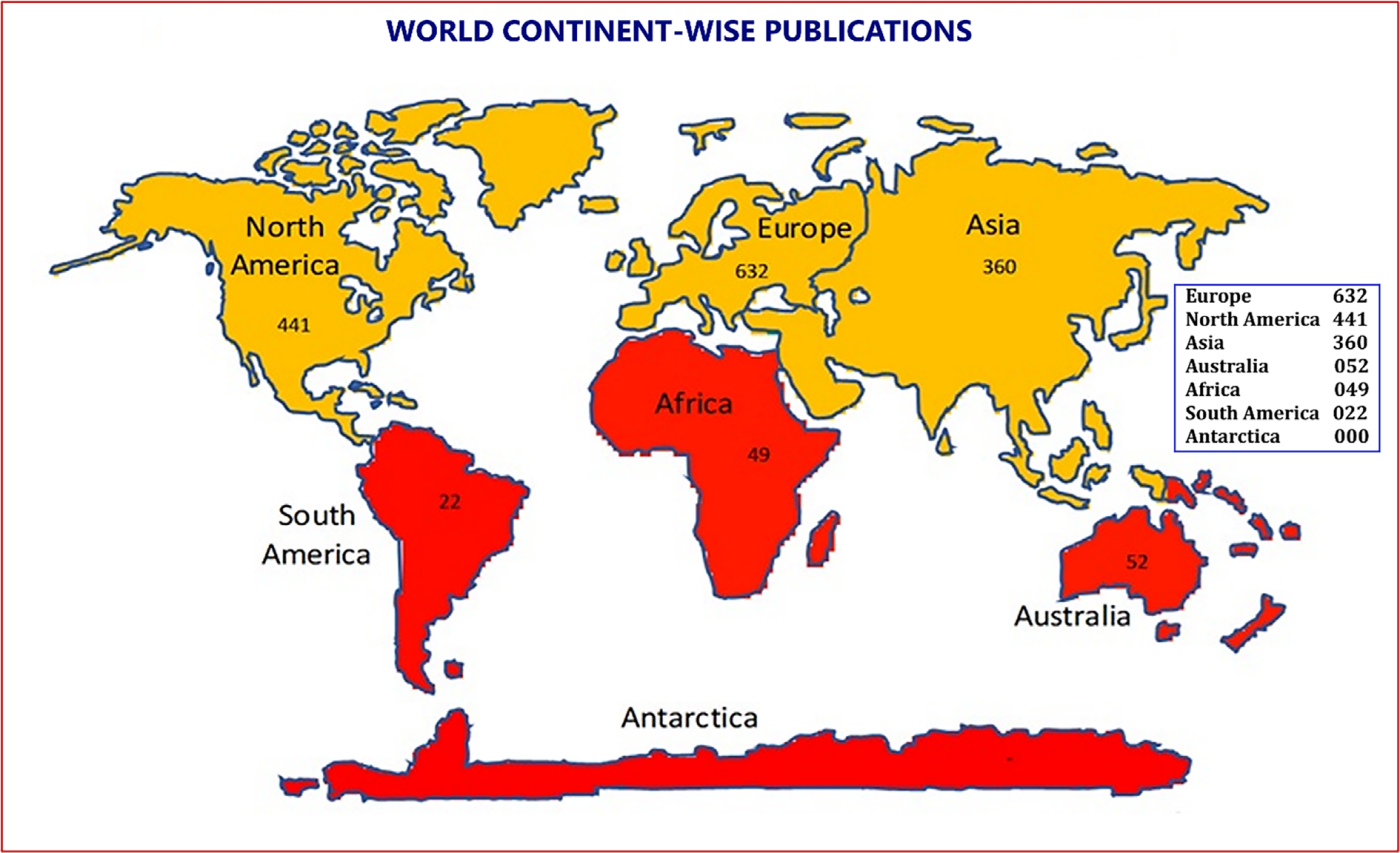
Continent-wise distribution of papers.

Europe leads in output with 632 publications and 9,445 citations (CPP 14.94; RCI 0.97) and shows the strongest international collaboration (25.47% ICP; 305 links). North America follows with 441 publications but achieves the highest impact, generating 9,318 citations (CPP 21.13; RCI 1.37) and 18 HCPs (Supplementary Table 5). Asia accounts for 360 publications, with strong funding (89 papers) but lower impact (CPP 10.58; RCI 0.68). Australia (52 publications; CPP 10.7; 28.85% ICP) and Africa (49 publications; CPP 14.6) show moderate output and collaboration, while South America has limited output (22 publications) but high collaboration (36.36% ICP) and low impact (RCI 0.54) (Figure 2).

### Author productivity and citation impact distribution

Author productivity is highly skewed (Table 2): 5,176 of 6,499 authors (79.6%) published only one paper, while just 18 authors produced six or more. Nelson J.D. leads with 10 papers (CPP 55.50, RCI 3.39, 3 HCPs), alongside Hunter S., Ceroni D., and Liu Y., with 10 papers each. Citation impact is concentrated among top authors, with several exceeding the overall CPP of 16.38; 2-paper authors show the highest average impact (CPP 18.78; RCI 1.15), while most- higher-output groups have RCIs below 1.00. Of 106,464 total citations, single-paper authors account for 83,880. Collaboration and funding are strongest among elite authors (1,410 funded papers overall), but vary widely, from high ICPs (e.g., Bonacorsi S. 33.3%) to none (e.g., Liu Y.).

**Table 2:** Most productive and impactful authors.

Author	Affiliation	TP	TC	CPP	HCP	FP	ICP	%ICP	RCI	Links
Hunter S.	Starship Hospital, Auckland, New Zealand	14	88	6.29	0	7	1	7.14	0.38	35
Nelson J.D.	UT Southwestern Medical School, Dallas, United States	10	555	55.50	3	0	0	0.00	3.39	12
Ceroni D.	Hôpitaux Universitaires de, Geneva, Switzerland	10	194	19.40	0	3	3	30.00	1.18	61
Liu Y.	Children's Hospital of Soochow University, Suzhou, China	10	30	3.00	0	10	0	0.00	0.18	62
Peltola H.	Helsingin Yliopisto, Helsinki, Finland	8	263	32.88	0	1	1	12.50	2.01	18
Ilharreborde B.	Hôpital Robert-Debré AP-HP, Paris, France	8	129	16.13	0	0	2	25.00	0.98	39
Crawford H.	University of Auckland, Auckland, New Zealand	8	85	10.63	0	6	0	0.00	0.65	29
Glorion C.	Hôpital Necker-Enfants Malades, Paris, France	6	271	45.17	1	0	1	16.67	2.76	29
Pääkkönen M.	Turun Yliopistollinen Keskussairaala, Turku, Finland	6	217	36.17	0	1	1	16.67	2.21	13
Lorrot M.	Hôpital Armand-Trousseau, Paris, France	6	116	19.33	0	1	1	16.67	1.18	43
Baker J.F.	Waikato Hospital, Hamilton, New Zealand	6	84	14.00	0	2	1	16.67	0.85	9
Li Y.	Hospital of Nanjing University of Chinese Medicine, Nanjing, China	6	80	13.33	0	3	1	16.67	0.81	34
Bonacorsi S.	INSERM, Paris, France	6	67	11.17	0	1	2	33.33	0.68	43
Mallet C.	Hôpital Robert-Debré AP-HP, Paris, France	6	61	10.17	0	1	1	16.67	0.62	36
Nygaard U.	Rigshospitalet, Copenhagen, Denmark	6	41	6.83	0	4	1	16.67	0.42	60
Wu X.	Henan Provincial People’s Hospital, Zhengzhou, China	6	28	4.67	0	5	1	16.67	0.28	42
Gouveia C.	Hospital de Dona Estefânia, Lisbon, Portugal	6	21	3.50	0	1	0	0.00	0.21	30
Wang X.	Children's Hospital of Soochow University, Suzhou, China	6	19	3.17	0	3	0	0.00	0.19	39
13 authors contributing 5 papers each		65	777	11.95	1	21	9	13.85	0.73	381
29 authors contributing 4 papers each		116	1591	13.72	1	39	18	15.52	0.84	848
76 authors contributing 3 papers each		228	3222	14.132	3	62	32	14.04	0.86	1341
390 authors contributing 2 papers each		780	14645	18.776	20	161	106	13.59	1.15	5081
5176 authors contributing 1 paper each		5176	83880	16.206	149	1078	707	13.66	0.99	42027
Total		6499	106464	16.38	178	1410	889	13.68	1.00	50312

TP = Total Publications; TC = Total Citations; CPP = Citations per Paper; HCP = Highly cited papers; FP = Funded Papers; ICP = International Collaborative Papers; RCI = Relative Citation Index.

### Institution-wise distribution

Institutional output is highly fragmented across 1,932 institutions, with 1,544 (79.9%) contributing only one paper. A small core of 14 institutions produced 147 publications, showing higher impact (CPP 20.06; RCI 1.27). The Children’s Hospital of Philadelphia is the most productive single institution (17 papers; CPP 17.47; RCI 1.11; 17.65% ICP). High impact is also observed seen among lower-volume institutions, notably Baylor College of Medicine (11 papers; CPP 52.64; RCI 3.33; 2 HCPs) and the University of Texas Health Science Center, Dallas, and Children’s Hospital Los Angeles (CPP >42; RCI ≥2.67). Strong collaboration enhances impact at institutions such as the Hospital for Sick Children, Toronto (44.44% ICP; CPP 28.11; RCI 1.78), whereas some high-output institutions from developing regions show low impact and limited collaboration (CPP ≤7.56; RCI <0.50) (Table 3).

**Table 3:** Most productive and impactful institutions.

Affiliations	TP	TC	CPP	HCP	FP	ICP	%ICP	RCI	Links
Children’s Hospital of Philadelphia, USA	17	297	17.47	0	2	3	17.65	1.11	43
University of Auckland, New Zealand	16	166	10.38	0	8	2	12.50	0.66	38
Baylor College of Medicine, Houston, USA	11	579	52.64	2	3	1	9.09	3.33	30
Boston Children’s Hospital, USA	11	169	15.36	0	2	3	27.27	0.97	25
Cincinnati Children’s Hospital, USA	10	123	12.30	0	2	2	20.00	0.78	46
PGIMER, Chandigarh, India	10	14	1.40	0	0	0	0.00	0.09	7
University of Texas Health Science Center, Dallas, USA	9	399	44.33	2	0	0	0.00	2.81	1
Children’s Hospital Los Angeles, USA	9	380	42.22	3	0	1	11.11	2.67	11
Hospital for Sick Children, Toronto, Canada	9	253	28.11	0	3	4	44.44	1.78	37
Case Western Reserve University School of Medicine, Cleveland, USA	9	175	19.44	1	1	0	0.00	1.23	13
University of Pennsylvania, USA	9	160	17.78	0	1	1	11.11	1.13	26
Harvard Medical School, Boston, USA	9	93	10.33	0	1	1	11.11	0.65	11
Robert Debre Hospital, Paris, France	9	73	8.11	0	0	3	33.33	0.51	12
AIIMS, New Delhi, India	9	68	7.56	0	1	1	11.11	0.48	3
6 institutes contributing 8 papers each	48	1121	23.35	2	6	7	14.58	1.48	116
5 institutes contributing 7 papers each	35	504	14.4	0	5	8	22.86	0.91	75
13 institutes contributing 6 papers each	78	1601	20.53	3	10	17	21.79	1.30	214
17 institutes contributing 5 papers each	85	1598	18.80	2	28	14	16.47	1.19	238
41 institutes contributing 4 papers each	164	2654	16.18	5	41	35	21.34	1.03	410
60 institutes contributing 3 papers each	180	2963	16.46	6	41	43	23.89	1.04	415
232 institutes contributing 2 papers each	464	8179	17.63	15	93	89	19.18	1.12	1108
1544 institute contributing 1 paper each	1544	21760	14.09	22	359	296	19.17	0.89	4355
Top 14 institutes contributing (5.36%)	147	2949	20.0612	8	24	22	14.97	1.27	303
Other 1918 institutes contributing (94.64%)	2598	40380	15.5427	55	583	509	19.59	0.98	6931
Total 1932 Institutes (100%)	2745	43329	15.7847	63	607	531	19.34	1.00	7234

TP = Total Publications; TC = Total Citations; CPP = Citations per Paper; HCP = Highly cited papers; FP = Funded Papers; ICP = International Collaborative Papers; RCI = Relative Citation Index.

### Journal-wise distribution of publications

Publications in PBJI are distributed across 514 journals, with the top 10 contributing 333 papers (24.38%) (Supplementary Table 6), and the remaining 504 journals accounting for 1,033 papers (75.62%). The *Journal of Pediatric Orthopaedics* leads with 95 papers and 2,618 citations (CPP 27.56; RCI 1.85; 5 HCPs), while the *Journal of Pediatric Orthopaedics Part B* publishes 58 papers but with a lower impact (CPP 10.50; RCI 0.71). High-impact, low-volume journals include *Pediatrics* (15 papers; CPP 47.80; RCI 3.21; 3 HCPs) and the *Journal of Bone and Joint Surgery (Series B)* (CPP 36.60; RCI 2.46). Specialty journals such as *Pediatric Radiology* and the *Pediatric Infectious Disease Journal* show balanced output and impact (CPP ~22–23; RCI >1), whereas high-volume practice-oriented outlets like *BMJ Case Reports* (45 papers) have very low impact (CPP 2.31; RCI 0.16).

### Keyword analysis

**Figure 3:**
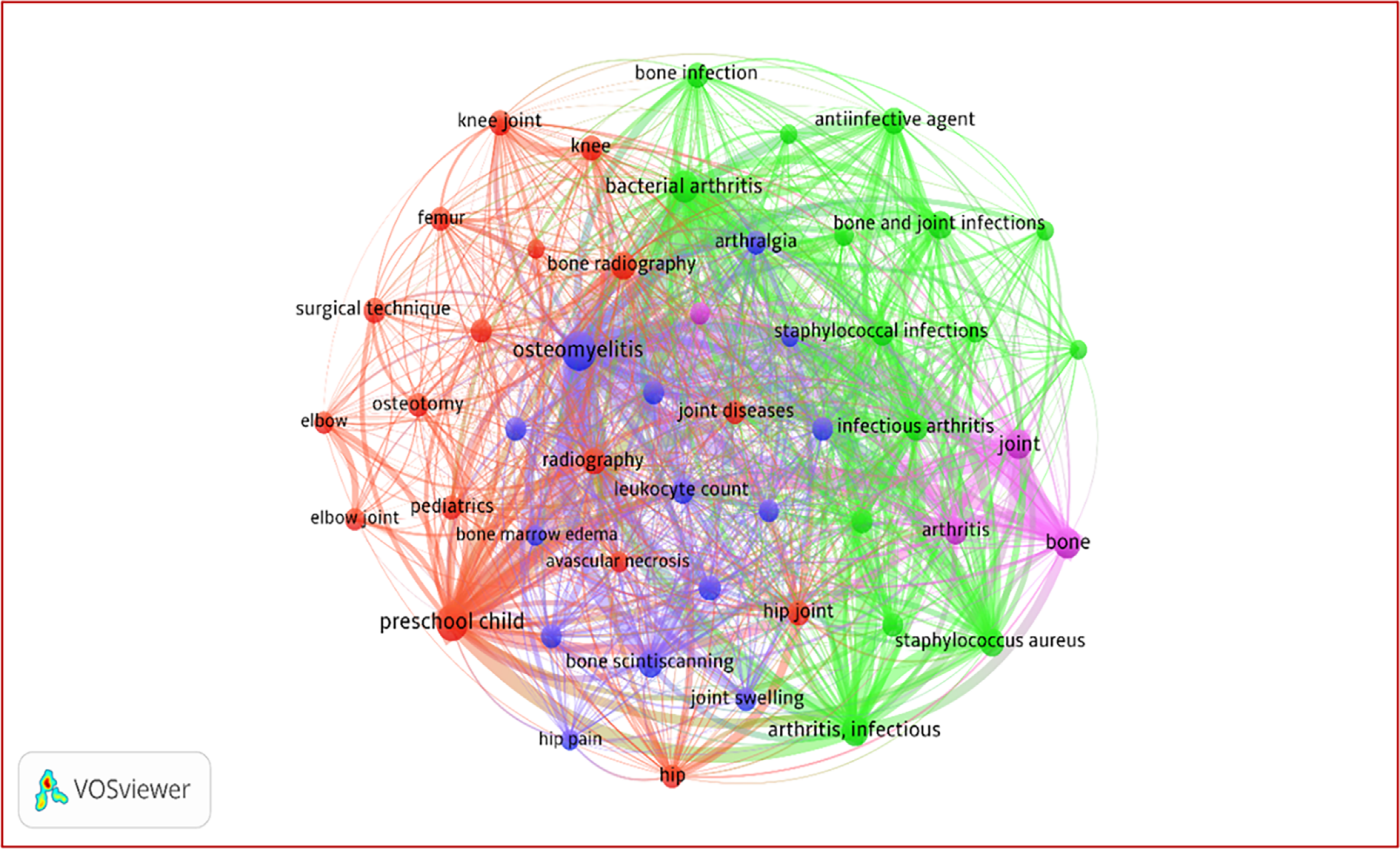
VOSviewer co-occurrence network of pediatric bone and joint infection research showing various clusters of the keywords.

From 10,153 keywords, the top 50 (minimum occurrence = 40) formed a dense co-occurrence network with 1,167 links and a total link strength (TLS) of 16,00 (Supplementary Table 7). The network clustered into four themes: Cluster 1 (17 keywords) centered on core diseases such as osteomyelitis and septic arthritis; Cluster 2 (15) focused on joint-specific and clinical features; Cluster 3 (14) addressed diagnostic, anatomical, and imaging aspects; and Cluster 4 (4) represented a small specialized niche (Figure 3).

### International co-authorship analysis

**Figure 4:**
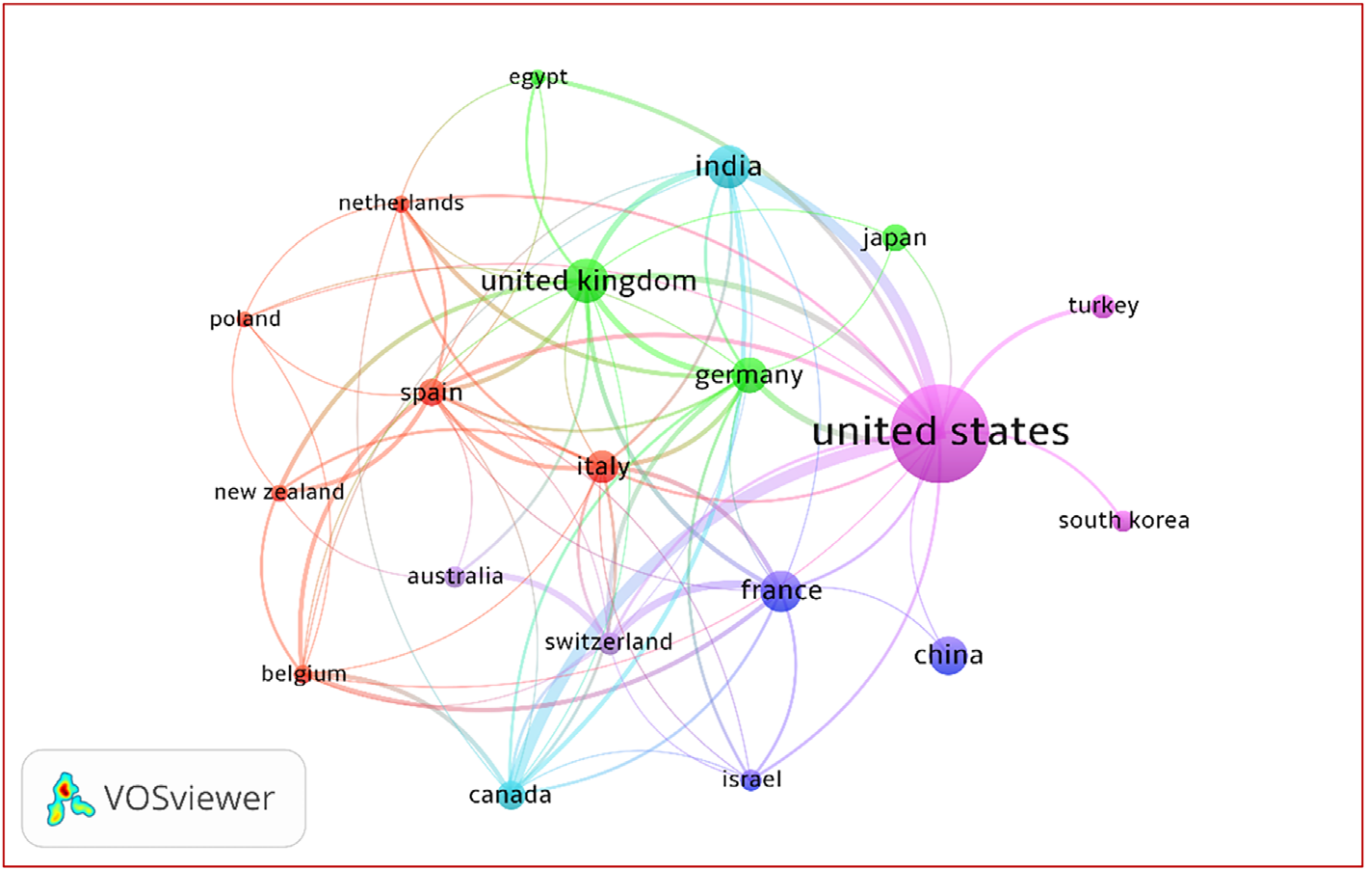
International co-authorship network map of pediatric bone and joint infection research.

Co-authorship analysis of 80 countries identified 20 meeting the ≥17-publication threshold, forming a collaboration network with 81 links and a TLS of 167. The network clustered into six groups: one large core cluster (6 countries), a second strong cluster (4), two intermediate clusters (3 each), and two small bilateral clusters (2 each) (Figure 4).

**Figure 5:**
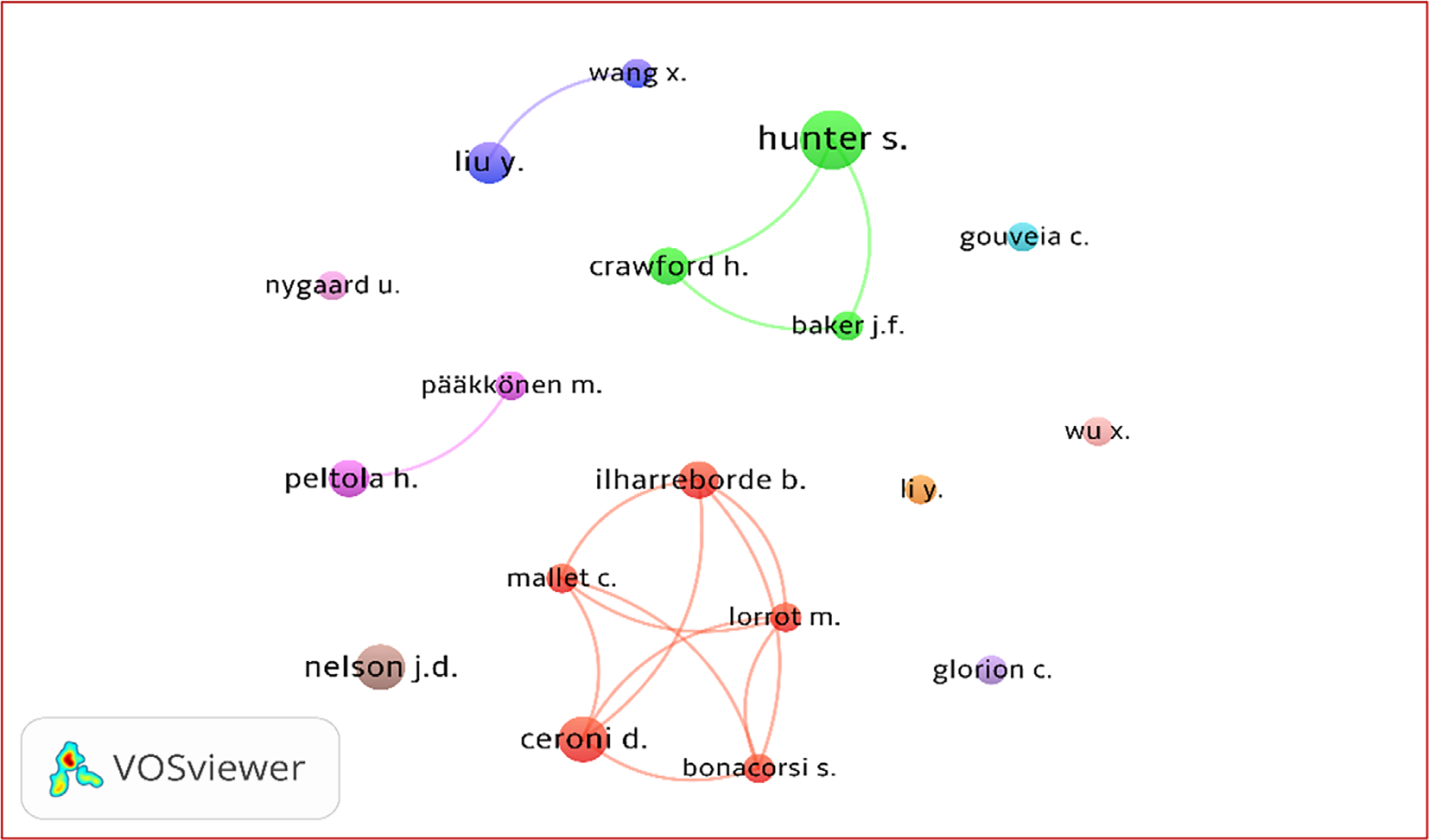
Co-authorship network of the top 18 authors in pediatric bone and joint infection research.

Co-authorship analysis of 5,146 authors identified 18 prolific contributors (≥6 publications) forming a selective collaboration network with 15 links and a TLS of 41. The network was structured into 10 clusters (Figure 5), with a core collaborative group of five authors – Bonacorsi S., Ceroni D., Ilharreborde B., Lorrot M., and Mallet C. – driving the strongest connections. A secondary cluster included Baker J.F., Crawford H., and Hunter S., while smaller two-author clusters and several single-author clusters indicate limited or independent collaboration among other leading authors in PBJI research.

### Co-authorship network analysis of the top 20 affiliations

**Supplementary Figure 1:**
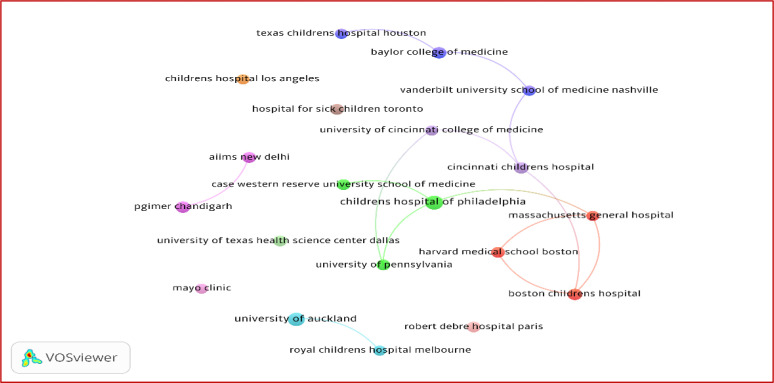
Co-authorship network of the top 20 affiliations in pediatric bone and joint infection research.

Affiliation-level co-authorship analysis identified 20 prolific institutions (≥8 publications) from 1,932 affiliations, forming a sparse collaboration network with 14 links and a TLS of 31. The network was highly fragmented into 20 clusters, with only three small clusters (3 affiliations each) showing cohesive collaboration, a few bilateral partnerships, and the majority of institutions operating independently or with weak inter-institutional links (Supplementary Figure 1).

### Highly cited papers

Table 4 highlights the top ten highly cited papers (130–301 citations) shaping PBJI research, published between 1978 and 2014, with reviews (4), original research articles (5), and one short survey contributing to high impact. The most cited paper is Almeida and Roberts’ UK-based review on bone involvement in sickle cell disease (301 citations), highlighting the influence of comprehensive syntheses [14]. The USA dominates contributions (5 papers), followed by the UK (2), France, Belgium, and Singapore.

**Table 4:** Top ten highly-cited papers in pediatric bone and joint infections.

Document Type	Authors	Affiliations	Title	Year	Source	Citations
Review	Almeida A., Roberts I. [14]	Hammersmith Hospital London, UK	Bone involvement in sickle cell disease	2005	*Br. J. Haematol.*	301
Review	Dodwell E.R. [1]	Hospital for Special Surgery, USA	Osteomyelitis and septic arthritis in children: Current concepts	2013	*Curr. Opin. Pediatr.*	189
Research Article	Dohin B., et al. [15]	Hopital Edouard Herriot Lyon; Universite Claude Bernard Lyon Villeurbanne, France	Pediatric bone and joint infections caused by panton-valentine leukocidin-positive Staphylococcus aureus	2007	*Pediatr. Infect. Dis. J.*	176
Research Article	Tetzlaff T.R., McCracken Jr. G.H., Nelson J.D. [16]	UT Southwestern Medical School Dallas, USA	Oral antibiotic therapy for skeletal infections of children. II. Therapy of osteomyelitis and suppurative arthritis	1978	*J. Pediatr.*	173
Short Survey	Teo H.E., Peh W.C. [17]	Kandang Kerbau Women’s and Children’s Hospital, Singapore	Skeletal tuberculosis in children	2004	*Pediatr. Radiol.*	159
Review	Jackson M.A., Nelson J.D. [18]	University of Texas Health Science Center Dallas, USA	Etiology and medical management of acute suppurative bone and joint infections in pediatric patients	1983	*J. Pediatr. Orthop.*	154
Research Article	Welkon C.J., et al. [19]	Temple University School of Medicine Philadelphia; St Christopher’s Hospital for Children, USA	Pyogenic arthritis in infants and children: A review of 95 cases	1986	*Pediatr. Infect. Dis.*	142
Research Article	Fabry G., Meire E. [20]	University Hospital Pellenberg, Belgium	Septic arthritis of the hip in children: Poor results after late and inadequate treatment	1983	*J. Pediatr. Orthop.*	139
Review	Kang S.N., et al. [21]	Royal London Hospital London; University of Nottingham Nottingham, UK	The management of septic arthritis in children: Systematic review of the english language literature	2009	*J. Bone Jt. Surg. Ser. B*	134
Research Article	Wang C.-L., et al. [22]	National Cheng Kung University & Hospital Tainan	Septic arthritis in children: Relationship of causative pathogens, complications, & outcome	2003	*J. Microbiol. Immunol. Infect.*	130

Core themes include osteomyelitis and septic arthritis management (Dodwell [1]; Jackson & Nelson [18]; Tetzlaff et al. [16]), pathogen-specific infections, particularly PVL-positive *Staphylococcus aureus* (Dohin et al. [15]), and special conditions such as skeletal tuberculosis (Teo & Peh [17]). Influential clinical journals such as the *British Journal of Haematology*, *Journal of Pediatrics*, *Journal of Pediatric Orthopaedics*, *Pediatric Infectious Disease Journal*, and *Journal of Bone and Joint Surgery (Series B)* serve as key dissemination platforms.

## Discussion

This comprehensive bibliometric review reveals a distinctly uneven but progressively structured landscape in pediatric bone and joint infection (PBJI) research. Across 1,556 publications analyzed, journal articles overwhelmingly dominate the literature, representing 82.4% of outputs (1,125 papers) with 15,156 citations (CPP = 16.44; RCI = 0.91), whereas review articles (156 papers) generate disproportionately greater influence (CPP = 31.67; RCI = 1.90) and account for 9 of 28 HCPs. English publications dominate emphatically (1,217 papers; 89.1% of outputs and 97.5% of citations; CPP = 19.19; RCI = 1.09), with all 28 HCPs written in English, indicating the language’s gatekeeping role in scientific influence. Citation distribution is heavily skewed: 18.4% of papers remain uncited, while a small core (86 papers; 6.3%) with >50 citations accounts for a substantial portion of total citations (CPP > 70), indicating concentrated impact among a minority of works. Research productivity and impact are concentrated in a small number of high-income countries, particularly the USA, the UK, France, and Germany, while many LMICs contribute modest output with limited citation impact, reflecting persistent global inequities in research capacity and dissemination.

Thematic and keyword co-occurrence analyses reveal a mature and conceptually integrated research landscape centered on osteomyelitis, septic arthritis, *Staphylococcus aureus*, and pediatric outcomes, with increasing emphasis on pathogen-specific disease (including MRSA and PVL-positive strains), advanced imaging, and optimized antibiotic strategies [23, 24]. This aligns with contemporary clinical literature showing a paradigm shift toward molecular diagnostics, particularly PCR-based detection of *Kingella kingae*, and evidence-based shortening of antibiotic courses with early oral switch protocols, without compromising outcomes [6, 7, 19, 25]. The concentration of HCPs between 2002 and 2009 reflects a pivotal period when these advances gained traction, reshaping diagnostic algorithms and management strategies [5, 6, 10]. The Pediatric Osteomyelitis Clinical Practice Guideline (2021) has emphasized improved risk stratification to predict adverse outcomes in children with osteomyelitis and has highlighted the role of clinical, laboratory, and imaging predictors to support timely, individualized management and better prognostication [26].

Collaboration analyses at the country, author, and institutional levels indicate that although international collaboration enhances impact, it remains selective and fragmented. A few countries and elite authors act as hubs, while many contributors operate in isolation or within localized networks. Notably, institutions and authors with higher international collaboration percentages often demonstrate superior citation impact, supporting prior evidence that cross-border partnerships amplify research visibility and influence [11, 17]. However, strong funding support, as observed in some regions, does not uniformly translate into higher impact, suggesting that research design, dissemination channels, and collaboration quality are equally critical.

Study’s Limitations: Firstly, it relies on a single bibliographic database (Scopus), which may underrepresent regional journals and non-English literature. Secondly, citation-based indicators inherently favor older publications and may not fully capture the influence of recent high-quality studies. Thirdly, bibliometric measures cannot assess clinical effectiveness or the real-world implementation of research findings. Finally, keyword and authorship analyses depend on indexing accuracy, which may introduce classification bias.

Future research should focus on longitudinal bibliometric updates to track emerging themes such as antimicrobial stewardship, artificial intelligence (AI) assisted diagnostics, and outcome prediction models. Greater emphasis is needed on PBJI research from low-resource and high-burden settings, where epidemiology, pathogen profiles, and access to diagnostics differ substantially. Strengthening international and interdisciplinary collaboration, promoting open-access dissemination, and integrating bibliometric insights with clinical outcome data will be essential to bridge current knowledge gaps.

This review demonstrates that PBJI research has evolved into a clinically focused, methodologically robust field driven by a small number of influential authors, institutions, and journals. Continued global collaboration and equitable research investment are crucial to ensure that advances in diagnosis and management translate into improved outcomes for children worldwide [1, 2, 6, 11].

## Conclusion

This bibliometric review demonstrates that pediatric bone and joint infection (PBJI) research is a mature yet unevenly distributed field, dominated by research articles, English-language publications, and contributions from a small number of high-income countries, authors, institutions, and journals. A small number of highly cited papers and leading research groups have a strong influence on the field, while many other contributors have a limited impact. Research themes are mainly focused on osteomyelitis, septic arthritis, pathogen-specific infections, and diagnostic and therapeutic advances. Strengthening international collaboration, improving visibility of research from low-resource settings, and fostering high-quality, clinically relevant studies are essential to achieving more equitable and impactful global PBJI research.

## Data Availability

The raw data is available from the corresponding Author.

## Supplementary materials

Supplementary Box 1
Search strategy used for the study.
Supplementary Table 1
Year-wise performance of publications with various indicators.
Supplementary Table 2
Document type-wise publications.
Supplementary Table 3
Language-wise distribution of publications.
Supplementary Table 4
Distribution of citations.
Supplementary Table 5
Continent-wise distribution of papers.
Supplementary Table 6
Most productive and impactful journals.
Supplementary Table 7
Top keywords.

The supplementary material of this article is available at https://www.sicot-j.org/10.1051/sicotj/2026024/olm.
