# Supplementary material for: Global research trends in pediatric bone and joint infections: A 50-year bibliometric analysis (1976–2025)
Source: SICOT J. 2026 May 27;12:34. doi: 10.1051/sicotj/2026024 (PMC13221163; doi:10.1051/sicotj/2026024)
Supplement: Supplementary file 1 — Search strategy used for the study. [file sicotj-12-34-s1.pdf]

((key (bone) and key (joint infection or osteomyelitis or septicarthritis))) and ((key (pediatr or paediatr* or child* or adolescent* or juvenile* or kid* or teenager* or neonate* or infant* or newborn* or kid or toddler or youth or youngster) and key (bone))) and pubyear > 1975 and pubyear < 2026 and (limit-to (exactkeyword , "child") or limit-to (exactkeyword , "adolescent") or limit-to (exactkeyword , "preschool child") or limit-to (exactkeyword , "child, preschool") or limit-to (exactkeyword , "infant") or limit-to (exactkeyword , "school child") or limit-to (exactkeyword , "infant, newborn") or limit-to (exactkeyword , "newborn") or limit-to (exactkeyword , "children") or limit-to (exactkeyword , "pediatrics") or limit-to (exactkeyword , "childhood disease") or limit-to (exactkeyword , "pediatric patient") or exclude (exactkeyword , "adult") or exclude (exactkeyword , "aged") or exclude (exactkeyword , "middle aged") or exclude (exactkeyword , "young adult") or exclude (exactkeyword , "aged, 80 and over") or exclude (exactkeyword , "very elderly") or exclude (exactkeyword , "middle age"))*

Box 1: Search Strategy used for the study
