# Supplementary material for: Global research trends in pediatric bone and joint infections: A 50-year bibliometric analysis (1976–2025)
Source: SICOT J. 2026 May 27;12:34. doi: 10.1051/sicotj/2026024 (PMC13221163; doi:10.1051/sicotj/2026024)
Supplement: Supplementary file 2 — Year-wise performance of publications with various indicators. [file sicotj-12-34-s2.pdf]

Supplementary Table 1: Year-wise performance of publications with various indicators

Year	TP	TC	CPP	TA	HCP	FP	ICP	%ICP	RCI
**1976**	3	38	19.00	8	0	0	0	0.00	0.85
**1977**	7	39	19.50	20	0	0	0	0.00	0.37
**1978**	17	253	25.30	34	1	0	0	0.00	1.00
**1979**	18	86	8.60	50	0	0	1	5.56	0.32
**1980**	15	317	35.22	46	1	0	0	0.00	1.42
**1981**	17	184	15.33	45	0	0	0	0.00	0.73
**1982**	15	188	18.80	46	0	0	1	6.67	0.84
**1983**	28	617	25.71	66	2	1	0	0.00	1.48
**1984**	20	268	15.76	64	0	0	0	0.00	0.90
**1985**	25	357	17.00	89	0	1	0	0.00	0.96
**1986**	28	775	31.00	83	3	0	2	7.14	1.86
**1987**	11	170	15.45	33	0	0	0	0.00	1.04
**1988**	6	52	13.00	21	0	0	0	0.00	0.58
**1989**	3	44	22.00	11	0	0	0	0.00	0.99
**1990**	7	55	9.17	23	0	0	0	0.00	0.53
**1991**	6	176	35.20	22	0	0	1	16.67	1.97
**1992**	10	187	18.70	29	0	0	0	0.00	1.26
**1993**	6	143	28.60	18	0	0	0	0.00	1.60
**1994**	5	193	38.60	17	0	0	0	0.00	2.60
**1995**	11	154	15.40	38	0	0	1	9.09	0.94
**1996**	4	57	14.25	10	0	0	1	25.00	0.96
**1997**	9	127	15.88	32	0	0	0	0.00	0.95
**1998**	15	363	27.92	52	1	1	2	13.33	1.63
**1999**	5	106	21.20	26	0	0	0	0.00	1.43

**2000**	12	371	37.10	41	0	0	0	0.00	2.08
**2001**	10	198	22.00	36	1	0	1	10.00	1.33
**2002**	13	272	27.20	46	0	0	0	0.00	1.41
**2003**	18	566	35.38	78	2	1	1	5.56	2.11
**2004**	12	424	35.33	48	1	0	1	8.33	2.38
**2005**	25	993	43.17	99	3	0	4	16.00	2.67
**2006**	19	620	34.44	65	2	0	0	0.00	2.19
**2007**	30	682	27.28	134	2	0	2	6.67	1.53
**2008**	25	340	14.78	108	0	0	3	12.00	0.91
**2009**	37	1035	30.44	169	3	3	1	2.70	1.88
**2010**	26	454	19.74	106	1	1	6	23.08	1.17
**2011**	37	725	21.97	146	1	1	0	0.00	1.32
**2012**	49	952	20.26	221	0	3	5	10.20	1.31
**2013**	51	1256	27.30	213	2	3	5	9.80	1.66
**2014**	43	907	25.19	274	1	2	10	23.26	1.42
**2015**	48	936	21.27	228	0	1	7	14.58	1.31
**2016**	57	716	14.04	297	0	4	8	14.04	0.84
**2017**	56	859	16.52	274	1	4	11	19.64	1.03
**2018**	57	647	12.94	327	0	5	8	14.04	0.76
**2019**	54	471	9.42	262	0	11	3	5.56	0.59
**2020**	60	710	13.65	405	0	10	7	11.67	0.80
**2021**	59	354	7.08	339	0	15	8	13.56	0.40
**2022**	67	425	7.73	393	0	30	11	16.42	0.43
**2023**	80	272	4.53	484	0	33	5	6.25	0.23
**2024**	65	145	3.92	381	0	23	7	10.77	0.15
**2025**	65	36	1.89	435	0	27	10	15.38	0.04

**Total**	**1366**	**20315**	**14.87**	**6492**	**28**	**180**	**133**	**9.74**	**1.00**

TP= Total Publications; TC= Total Citations; CPP= Citations per Paper; TA= Total Authors; HCP= Highly cited papers; FP= Funded Papers; ICP= International Collaborative Papers; RCI= Relative Citation Index
