# Supplementary material for: Global research trends in pediatric bone and joint infections: A 50-year bibliometric analysis (1976–2025)
Source: SICOT J. 2026 May 27;12:34. doi: 10.1051/sicotj/2026024 (PMC13221163; doi:10.1051/sicotj/2026024)
Supplement: Supplementary file 3 — Document type-wise publications. [file sicotj-12-34-s3.pdf]

**Supplementary Table 2: Document type-wise publications**

| Document Type | TP | TC | CPP | TA | HCP | FP | ICP | %ICP | RCI |
|---|---|---|---|---|---|---|---|---|---|
| **Research Article** | 1125 | 15156 | 16.44 | 5525 | 18 | 155 | 104 | 9.24 | 0.91 |
| **Review** | 156 | 4402 | 31.67 | 642 | 9 | 18 | 19 | 12.18 | 1.90 |
| **Letter** | 30 | 158 | 7.18 | 146 | 0 | 4 | 5 | 16.67 | 0.35 |
| **Note** | 18 | 16 | 2.67 | 61 | 0 | 1 | 0 | 0.00 | 0.06 |
| **Conference Paper** | 15 | 228 | 19.00 | 61 | 0 | 1 | 0 | 0.00 | 1.02 |
| **Editorial** | 12 | 22 | 4.40 | 23 | 0 | 1 | 2 | 16.67 | 0.12 |
| **Short Survey** | 7 | 317 | 45.29 | 16 | 1 | 0 | 0 | 0.00 | 3.05 |
| **Retracted** | 1 | 10 | 10.00 | 11 | 0 | 0 | 1 | 100.00 | 0.67 |
| **Book** | 1 | 6 | 6.00 | 4 | 0 | 0 | 1 | 100.00 | 0.40 |
| **Book Chapter** | 1 | 0 | 0.00 | 3 | 0 | 0 | 1 | 100.00 | 0.00 |
| **Total** | **1366** | **20315** | **14.87** | **6492** | **28** | **180** | **133** | **9.74** | **1.00** |

*TP= Total Publications; TC= Total Citations; CPP= Citations per Paper; TA= Total Authors; HCP= Highly cited papers; FP= Funded Papers; ICP= International Collaborative Papers; RCI= Relative Citation Index*
