# Supplementary material for: Global research trends in pediatric bone and joint infections: A 50-year bibliometric analysis (1976–2025)
Source: SICOT J. 2026 May 27;12:34. doi: 10.1051/sicotj/2026024 (PMC13221163; doi:10.1051/sicotj/2026024)
Supplement: Supplementary file 4 — Language-wise distribution of publications. [file sicotj-12-34-s4.pdf]

Supplementary Table 3: Language-wise distribution of publications

Language	TP	TC	CPP	TA	HCP	FP	ICP	%ICP	RCI
**English**	1217	19803	19.19	5821	28	177	131	10.76	1.09
**French**	50	175	5.65	229	0	0	1	2.00	0.24
**German**	32	200	9.09	97	0	0	0	0.00	0.42
**Spanish**	18	64	7.11	161	0	0	0	0.00	0.24
**Russian**	17	11	2.20	45	0	1	0	0.00	0.04
**Chinese**	6	5	1.25	34	0	2	0	0.00	0.06
**Italian**	4	6	6.00	17	0	0	0	0.00	0.10
**Dutch**	4	3	1.50	16	0	0	0	0.00	0.05
**Portuguese**	3	36	12.00	9	0	0	0	0.00	0.81
**Polish**	3	3	3.00	10	0	0	0	0.00	0.07
**Czech**	2	5	2.50	7	0	0	1	50.00	0.17
**Hebrew**	2	1	1.00	6	0	0	0	0.00	0.03
**Japanese**	2	0	0.00	12	0	0	0	0.00	0.00
**Slovak**	2	0	0.00	6	0	0	0	0.00	0.00
**Danish**	1	2	2.00	4	0	0	0	0.00	0.13
**Korean**	1	1	1.00	7	0	0	0	0.00	0.07
**Catalan**	1	0	0.00	7	0	0	0	0.00	0.00
**Swedish**	1	0	0.00	4	0	0	0	0.00	0.00
**Total**	**1366**	**20315**	**14.87**	**6492**	**28**	**180**	**133**	**9.74**	**1.00**

TP= Total Publications; TC= Total Citations; CPP= Citations per Paper; TA= Total Authors; HCP= Highly cited papers; FP= Funded Papers; ICP= International Collaborative Papers; RCI= Relative Citation Index
