# Supplementary material for: Global research trends in pediatric bone and joint infections: A 50-year bibliometric analysis (1976–2025)
Source: SICOT J. 2026 May 27;12:34. doi: 10.1051/sicotj/2026024 (PMC13221163; doi:10.1051/sicotj/2026024)
Supplement: Supplementary file 5 — Distribution of citations. [file sicotj-12-34-s5.pdf]

Supplementary Table 4: Distribution of Citations

Citation Slab	TP	Cumulative TP	TC	Cumulative TC	CPP
**0**	251	251	0	0	0
**1-10**	616	867	2573	2573	4.18
**11-25**	261	1128	4408	6981	16.89
**26-50**	152	1280	5410	12391	35.59
**51-100**	58	1338	4073	16464	70.22
**101-200**	26	1364	3344	19808	128.62
**201-301**	2	1366	507	20315	253.5
**Total**	**1366**		**20315**		**14.87**

TP= Total Publications; TC= Total Citations; CPP= Citations per Paper
