# Supplementary material for: Global research trends in pediatric bone and joint infections: A 50-year bibliometric analysis (1976–2025)
Source: SICOT J. 2026 May 27;12:34. doi: 10.1051/sicotj/2026024 (PMC13221163; doi:10.1051/sicotj/2026024)
Supplement: Supplementary file 6 — Continent-wise distribution of papers. [file sicotj-12-34-s6.pdf]

Supplementary Table 5: Continent-wise distribution of papers

Continent	TP	TC	CPP	TA	HCP	FP	ICP	%ICP	RCI	Links
**Europe**	632	9445	14.94	3701	14	94	161	25.47	0.97	305
**North America**	441	9318	21.13	1939	18	44	72	16.33	1.37	116
**Asia**	360	3808	10.58	1875	3	89	59	16.39	0.68	98
**Australia**	52	761	14.63	236	0	13	15	28.85	0.95	26
**Africa**	49	524	10.69	249	0	2	12	24.49	0.69	19
**South America**	22	184	8.36	134	0	5	8	36.36	0.54	18
**Antarctica**	00	00	0.00	00	00	00	00	0.00	0.00	00
**Total**	**1556**	**24040**	**15.45**	**8134**	**35**	**247**	**327**	**21.02**	**1.00**	**582**

TP= Total Publications; TC= Total Citations; CPP= Citations per Paper; TA= Total Authors; HCP= Highly cited papers; FP= Funded Papers; ICP= International Collaborative Papers; RCI= Relative Citation Index
