# Supplementary material for: Global research trends in pediatric bone and joint infections: A 50-year bibliometric analysis (1976–2025)
Source: SICOT J. 2026 May 27;12:34. doi: 10.1051/sicotj/2026024 (PMC13221163; doi:10.1051/sicotj/2026024)
Supplement: Supplementary file 7 — Most productive and impactful journals. [file sicotj-12-34-s7.pdf]

Supplementary Table 6: Most productive and impactful Journals

Journal Title	TP	TC	CPP	%TP	TA	HCP	FP	ICP	%ICP	RCI
Journal of Pediatric Orthopaedics	95	2618	27.56	6.95	423	5	5	11	11.58	1.85
Journal of Pediatric Orthopaedics Part B	58	609	10.50	4.25	284	0	5	11	18.97	0.71
BMJ Case Reports	45	104	2.31	3.29	164	0	1	2	4.44	0.16
Pediatric Radiology	32	732	22.88	2.34	133	1	4	5	15.63	1.54
Pediatric Infectious Disease Journal	23	507	22.04	1.68	132	1	4	4	17.39	1.48
Clinical Orthopaedics and Related Research	19	346	18.21	1.39	68	0	1	1	5.26	1.22
Injury	16	346	21.63	1.17	82	0	0	2	12.50	1.45
Pediatrics	15	717	47.80	1.10	67	3	2	0	0.00	3.21
Journal of Bone and Joint Surgery - Series B	15	549	36.60	1.10	47	1	0	2	13.33	2.46
International Orthopaedics	15	321	21.40	1.10	69	0	2	3	20.00	1.44
Top 10 Sources (shares 24.38%)	333	6849	20.57	24.38	1469	11	24	41	12.31	1.00
Total of 504 Sources (Shares 75.62)	1033	13466	13.04	75.62	5030	17	156	92	8.91	1.00
Total of 514 sources	**1366**	**20315**	**14.87**	**100.00**	**6499**	**28**	**180**	**133**	**9.74**	**1.00**

TP= Total Publications; TC= Total Citations; CPP= Citations per Paper; HCP= Highly cited papers; FP= Funded Papers; ICP= International Collaborative Papers; RCI= Relative Citation Index
