# Supplementary material for: Global research trends in pediatric bone and joint infections: A 50-year bibliometric analysis (1976–2025)
Source: SICOT J. 2026 May 27;12:34. doi: 10.1051/sicotj/2026024 (PMC13221163; doi:10.1051/sicotj/2026024)
Supplement: Supplementary file 8 — Top keywords. [file sicotj-12-34-s8.pdf]

Supplementary Table 7: Top Keywords

Keyword	Occ.	Clusters	Links	TLS	Keyword	Occ.	Clusters	Links	TLS
preschool child	485	1	49	2037	bone infection	112	2	49	555
bone radiography	167	1	49	681	staphylococcus infection	82	2	47	572
radiography	139	1	49	615	childhood disease	72	2	49	395
surgical technique	112	1	46	299	kingella kingae	59	2	44	425
hip joint	108	1	49	630	haemophilus influenzae	44	2	42	319
knee	103	1	49	494	hematogenous osteomyelitis	42	2	46	269
knee joint	102	1	44	452	streptococcus pneumoniae	40	2	47	288
osteotomy	97	1	43	317	osteomyelitis	640	3	49	3161
hip	92	1	48	563	bone scintiscanning	157	3	49	917
pediatrics	85	1	48	365	leukocyte count	109	3	49	685
fracture	85	1	46	312	joint effusion	100	3	48	527
joint diseases	83	1	46	361	arthralgia	94	3	49	479
sacroiliac joint	82	1	41	387	septic arthritis	86	3	48	630
femur	80	1	48	362	joint swelling	85	3	45	357
elbow	75	1	45	297	chronic osteomyelitis	79	3	48	422
elbow joint	68	1	34	222	juvenile rheumatoid arthritis	79	3	48	387
avascular necrosis	57	1	47	323	bone biopsy	79	3	44	370
bone necrosis	44	1	45	208	pediatric patient	66	3	47	307
bacterial arthritis	294	2	49	1882	hip pain	58	3	45	318
arthritis, infectious	223	2	49	1587	bone marrow edema	52	3	46	287
staphylococcus aureus	180	2	48	1215	sacroiliitis	41	3	40	236
bone and joint infections	157	2	47	766	bone	231	4	49	1068
infectious	14	2	49	10	joint	20	4	47	96

arthritis	4			58		4			8
antiinfective agent	128	2	49	736	arthritis	138	4	49	731
staphylococcal infections	121	2	47	796	bacterial infection	65	4	46	364

Occ.: Occurrences; TLS: Total Link Strength
